# Whole-genome sequences of influenza A(H3N2) viruses isolated from Brazilian patients with mild illness during the 2014 season

**DOI:** 10.1590/0074-02760140412

**Published:** 2015-02

**Authors:** Paola Cristina Resende, Fernando Couto Motta/+, Priscila Silva Born, Milene Miranda, Marilda M Siqueira

**Affiliations:** Laboratório de Vírus Respiratórios e do Sarampo, Instituto Oswaldo Cruz-Fiocruz, Rio de Janeiro, RJ, Brasil

**Keywords:** influenza, H3N2, Ion Torrent, NGS

## Abstract

The influenza A(H3N2) virus has circulated worldwide for almost five decades and is the dominant subtype in most seasonal influenza epidemics, as occurred in the 2014 season in South America. In this study we evaluate five whole genome sequences of influenza A(H3N2) viruses detected in patients with mild illness collected from January-March 2014. To sequence the genomes, a new generation sequencing (NGS) protocol was performed using the Ion Torrent PGM platform. In addition to analysing the common genes, haemagglutinin, neuraminidase and matrix, our work also comprised internal genes. This was the first report of a whole genome analysis with Brazilian influenza A(H3N2) samples. Considerable amino acid variability was encountered in all gene segments, demonstrating the importance of studying the internal genes. NGS of whole genomes in this study will facilitate deeper virus characterisation, contributing to the improvement of influenza strain surveillance in Brazil.

Influenza A(H3N2) viruses have circulated worldwide since 1968 causing high rates of morbidity and mortality (WHO 2014a ). During the 2014 season in the Southern Hemisphere, especially in South America's South Cone and Brazil, the subtype A(H3N2) was predominant among circulating influenza subtypes (PAHO 2014, Silva et al. 2014). Due to its important impact on public health, the constant improvement of methodologies for virus characterisation and evaluation of the match between circulating viruses and vaccine strains is necessary. As part of the efforts to characterise influenza A(H3N2) viruses circulating in Brazil, we sequenced the whole genome of five samples with a new generation sequencing (NGS) protocol. This allowed for a comparison among the Brazilian sequences and the World Health Organization (WHO) vaccine recommended strains for the 2013-2014 period in addition to implementing a new genomic tool in the Brazilian surveillance network.

The Brazilian samples were collected from patients with mild infection from January-March 2014 in the southern [state of Paraná (PR), lat: -25.252089/long: -52.021542, and the state Santa Catarina, lat: -27.242339/long: -50.218856] and southeastern (state of Rio de Janeiro, lat: -22.908300/long: -43.197077) regions. Viral RNA was extracted directly from one clinical sample and from four isolates after one passage in Madin-Darby canine kidney (MDCK) cells using QIAmp Viral RNA Mini Kit (Qiagen, Germany) following the manufacturer's instructions. All eight influenza gene segments were amplified at Respiratory and Measles Viruses Laboratory (Oswaldo Cruz Foundation) with PathAmp(tm) FluA Reagents (Life Technologies, USA), a two steps multiplex reverse transcription-polymerase chain reaction (PCR) rendering a six-band profile on a standard 2% agarose gel. All subsequent bench work for massive parallel sequencing was carried out at Thermo Fisher Scientific Brazil's headquarters laboratory in the state of São Paulo. In summary, the quality, quantity and integrity of total DNA pools were evaluated using Qubit^(r)^ dsDNA HS Assay Kit and Qubit 2.0 fluorometer (Thermo Fisher Scientific, DE). After that, the genome segments from each sample were enzymatically cleaved in fragments of approximately 250 base pairs (bp) and a barcode was linked to each pool of genome fragments, forming the libraries. After the emulsion PCR, DNA-positive beads were recovered, enriched and subjected to ion semiconductor sequencing using a 314 chip on the Ion Torrent sequencer (Thermo Fisher Scientific, DE). The reads obtained were subjected to quality filtering using a specific plug-in available from <ioncommunity.lifetechnologies.com/docs/DOC-7384>. All reads with Q score ≥ 20 were aligned with reference sequences, A/Texas/50/2012 (GISAID EPI ISL 164401), used in the 2014 epidemic, and A/Switzerland/9715293/2013 (GISAID EPI ISL 166310), the vaccine strain elected for 2015 season (WHO 2013, 2014b). The comparison with reference genomes and posterior analysis were conducted using Geneious software v.6.1.6 (Biomatters).

The concatenated whole genome of influenza A(H3N2) strains was 13,138 bp in length, with a 43.33% G+C content. The length of segments ranged from ~2,300 for the polymerase basic (PB)2 gene to ~900 bases for the non-structural (NS) gene. The sequencing protocol generated high coverages for each sample and the average coverage for each genomic segment ranged from 144-968 reads. The quality of sequencing was high for the majority of the genomic segments, however, two samples presented low coverage for the polymerase acidic (PA) and PB1 genes, yielding partially sequenced fragments.

Analysing the theoretical translation of whole genomes, we detected an average of 23 amino acid substitutions in the 2014 Brazilian A(H3N2) sequences in comparison to the genome of the A/Texas/50/2012 vaccine strain. Amino acid substitutions were found in all genes, especially in those that code for the surface glycoproteins, haemagglutinin (HA) and neuraminidase (NA). The HA gene accumulated more substitutions (5-8) in comparison to the other genes, such as N144A, R158G, N161S, P214S, V363M. No differences were observed between the clinical and isolated viruses from the A/RS/23/2014 sample, however it was the virus that presented the most substitutions among analysed samples, bearing the changes N22S, A388S and A492V, which were not observed in the other samples. Phylogenetically, the HA gene sequences from the Brazilian samples were included inside the group 3C.3, a distinct group from A/Texas/50/2012 at group 3C.2 according to the report sent to WHO for Vaccine Recommendation meeting (SVS 2014). The NA gene substitution H150R was detected in all samples when compared to the A/Texas/50/2012 vaccine strain. In addition, the isolated samples (A/PR/87/2014 and A/PR/88/2014) from PR presented I222V, N329T and D339N. The mutations found in the NA genes were not associated with decreased sensitivity to the antiviral oseltamivir. It was noted that when the NA sequences from isolated and non-isolated A/RS/23/2014 samples were compared, the residue D151 was observed in the non-isolated sample while the polymorphism D/N151 was observed in the MDCK isolated virus. In addition, virus from the isolated sample also presented the polymorphism I73I/T. These findings suggest that viruses isolated in MDCK cells might promote a viral pressure to adapt the virus as in agreement with a previous study (Tamura et al. 2013). The matrix gene only presented the substitution E290K when compared to the vaccine strain. Assessing the internal genes, we found several mutations, namely PB2 (V122I, L384F, E472D, R630K and D740N), PB1 (A83V, R215K and D464D), PA (V323I, V602I, N614S and I621V), NS (E26K, R44K, K67R, Q193H, K217R, R227G and the R248 stop codon) and for the nucleoprotein gene (S69S/P, V100I, V197I and T472A).

Concerning the WHO vaccine recommendations for the 2015 influenza season (WHO 2014b), the new prototype A/Switzerland/9715293/2013 (H3N2)-like virus was genetically closer to the Brazilian sequences than A/Texas/50/2012. This assumption is based mainly on the changes N144A, R158G and P214S of the HA epitopes A, B and D, respectively, diminishing the distance among the vaccine prototype and the more recent circulating strains. The information obtained with whole-genome sequencing of the A(H3N2) isolates in this study is important for strain surveillance, forecasts the emergence of new variants and corroborates the decision to change the vaccine strain for the 2015 epidemic season.

*Nucleotide sequence accessions* - The whole-genome sequences of the Brazilian influenza A(H3N2) samples from 2014 were deposited in the GISAID database (Global Initiative on Sharing All Influenza Data) under the accessions listed in Table.

**TABLE.** Accessions in the Global Initiative on Sharing All Influenza Data database

	Accessions				
Gene	A/RS/23/2014^*a*^	A/RS/23/2014	A/RJ/34/2014	A/PR/87/2014	A/PR/88/2014
PB2	EPI543520	EPI543528	EPI543542	EPI543548	EPI543556
PB1	EPI543521	EPI543529	-	EPI543549	EPI543557
PA	EPI543522	EPI543530	-	EPI543550	-
HA	EPI543523	EPI543531	EPI543543	EPI543551	EPI543558
NP	EPI543524	EPI543532	EPI543544	EPI543552	EPI543559
NA	EPI543525	EPI543533	EPI543545	EPI543553	EPI543560
M	EPI543526	EPI543534	EPI543546	EPI543554	EPI543561
NS	EPI543527	EPI543535	EPI543547	EPI543555	EPI543562

*a*: original specimen; HA: haemagglutinin; M: matrix; NA: neuraminidase; NP: nucleoprotein; NS: non-structural; PA: polymerase acidic; PB: polymerase basic; PR: state of Paraná; RJ: state of Rio de Janeiro; RS: state of Rio Grande do Sul.
